# The Efficacy of Radiotherapy in the Treatment of Hepatocellular Carcinoma with Distant Organ Metastasis

**DOI:** 10.1155/2021/5190611

**Published:** 2021-11-17

**Authors:** Lei Chen, Zhiwen Wang, Songlin Song, Tao Sun, Yanqiao Ren, Weihua Zhang, Mingfu Wang, Yiming Liu, Chuansheng Zheng

**Affiliations:** ^1^Department of Radiology, Union Hospital, Tongji Medical College, Huazhong University of Science and Technology, Wuhan 430022, China; ^2^Department of Interventional Radiology, Union Hospital, Tongji Medical College, Huazhong University of Science and Technology, Wuhan 430022, China; ^3^Department of Cardiology, Union Hospital, Tongji Medical College, Huazhong University of Science and Technology, Wuhan 430022, China; ^4^Department of Radiology, The Third People's Hospital of Hubei Province, Wuhan 430033, China

## Abstract

**Background:**

Recently, radiotherapy has been used in the treatment of hepatocellular carcinoma (HCC). However, there is no study analyzing the efficacy of radiotherapy in cases of advanced HCC. The objective of this investigation was to determine the efficacy of radiotherapy in patients with HCC invading distant organs.

**Methods:**

The data of 2342 patients diagnosed between 2010 and 2015 with HCC invading distant organs were extracted from the SEER database. Propensity score matching (PSM) was used to reduce selection bias.

**Results:**

Before PSM, the median overall survival (mOS) and median cancer-specific survival (mCSS) in the radiotherapy group (mOS = 5 months, 95% CI: 4.5–5.5; mCSS = 5 months, 95% CI: 4.4–5.6) were longer than those in the nonradiotherapy group (mOS = 3 months, 95% CI: 2.8–3.2; mCSS = 3 months, 95% CI: 2.8–3.2; both *P* < 0.001). After PSM, mOS in the radiotherapy group (5 months, 95% CI: 4.5–5.5) was longer than that in the nonradiotherapy group (3 months, 95% CI: 2.6–3.4; *P* < 0.001), and the mCSS in the radiotherapy group (5 months, 95% CI: 4.4–5.6) was longer than that in the nonradiotherapy group (3 months, 95% CI: 2.6–3.4; *P* < 0.001). Before PSM, the multivariate analysis showed that all-cause and cancer-specific mortality rates were higher in the nonradiotherapy group than in the radiotherapy group. The adjusted Cox regression analysis for subgroups showed that, in the nonradiotherapy group, patients with bone metastases and multiorgan metastases had a worse survival than those in the radiotherapy group.

**Conclusion:**

HCC patients with metastases to distant organs obtain survival benefit from radiotherapy, particularly patients with bone metastases and multiorgan metastases.

## 1. Introduction

Hepatocellular carcinoma (HCC) is one of the most common cancers with one of the highest fatality rates [1]. Patients with early HCC can get survival benefits from transplantation, surgery, or ablation [2–5]. Transarterial chemoembolization (TACE) can prolong the overall survival of patients with intermediate HCC, and some studies have shown the survival benefits of TACE in patients with advanced HCC [6, 7]. However, due to the lack of high-level evidence, TACE is currently not considered the first-line treatment for advanced HCC [8]. Sorafenib and lenvatinib are recommended for this category of patients, but these drugs are expensive, and the response rate is low [8–10]. Although in the last year a progress in early diagnosis of HCC has been reached, the changed scenario characterized by emerging etiologies such as metabolic causes of cirrhosis has led to a high rate of patients who receive HCC diagnosis in advance stages characterized by extrahepatic spread, as recently reported [11]. This changed scenario needs to enhance treatment strategies for advanced HCC, including radiotherapy.

Radiotherapy includes external and external radiotherapy, and both forms are being used in the treatment of several types of solid tumors [12–15]. Lin J. and coworkers reported that HCC patients with portal vein tumor thrombus (PVTT) could obtain survival benefits from iodine-125 seeds scaffold [16]. Another meta-analysis yielded similar results [17]. Some studies showed that external radiotherapy prolonged the overall survival (OS) of patients with different types of tumors that invaded distant organs [18–20]. A random controlled trial documented that external radiotherapy could prolong the median OS of patients with oligometastasis from different primary tumors [21]. However, for several decades, radiotherapy has not been recommended for the treatment of HCC because the radiation could damage healthy liver tissue. However, with the advancement of technology, the accuracy of external radiotherapy is more precise, allowing the clinicians to avoid damage to the normal liver tissue around the tumor. Several recent studies demonstrated that patients with HCC could gain survival benefits from radiotherapy [22–24].

The survival prognosis of patients with HCC that invades distant organs is dismal. Although radiotherapy benefits patients with different primary tumors invading distant organs, there are no studies addressing the question of whether patients with HCC metastasizing to distant organs can obtain survival benefits from radiotherapy. Thus, we compared the efficacy of radiotherapy with other treatments in HCC patients with the tumor invading distant organs.

## 2. Materials and Methods

### 2.1. Patient selection

Data used in the study originated from the Surveillance, Epidemiology, and End Results (SEER) database and were extracted using the SEER∗Stat software. The SEER database collects data on cancer cases from various locations and sources throughout the United States and includes approximately 28% of the United States population. The present analysis utilized the SEER data on patients diagnosed with HCC. The study was approved by the institutional Ethics Committee. The written informed consent was waived, since anonymized data were obtained from the national database.

**Figure 1:**
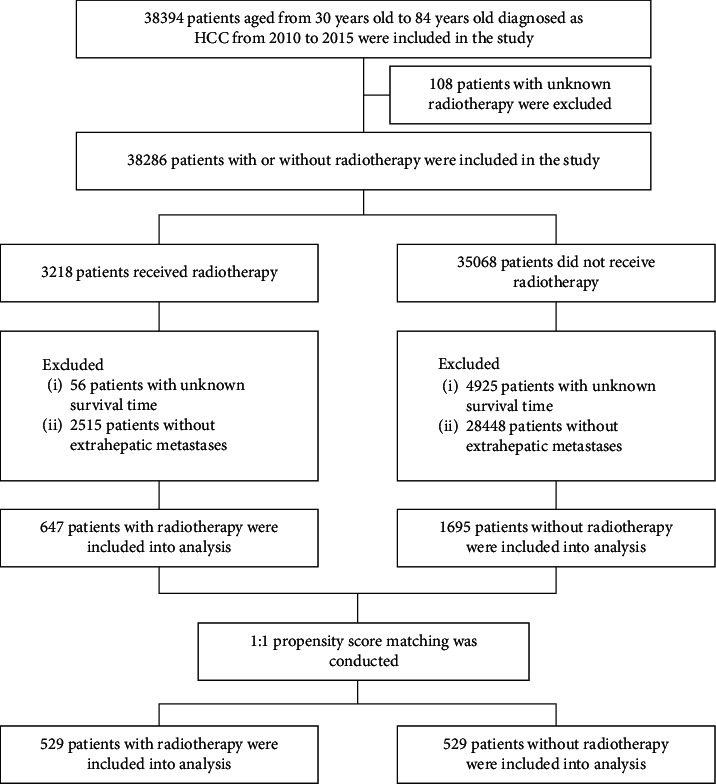
The flowchart of patient selection.

The inclusion criteria were as follows: (1) patients diagnosed as HCC (International Classification of Disease for Oncology, Third Edition (ICD-O-3), histology codes 8170/3–8175/3) between 2010 and 2015; (2) patients aged between 30 and 84 years; (3) patients having extrahepatic metastases (including multiorgan invasion); (4) patients for whom the information on radiotherapy treatment (yes or no) was available; and (5) patients having a known survival time (those with survival codes 0 and 999 were excluded) (Figure 1).

### 2.2. Definition of the Endpoints

The endpoints of the study were overall survival (OS) and cancer-specific survival (CSS). OS was defined as the interval from the time patients were diagnosed with HCC to the time of death caused by any reason. CSS was defined as the interval from the time of HCC diagnosis to the time of death caused by the cancer.

### 2.3. Statistical Analysis

The study included twelve baseline factors, and the continuous variables were converted to categorical variables. Chi-square test and Fisher's test were used to compare the difference of baseline factors between the radiotherapy and nonradiotherapy groups. The survival curves were plotted using the Kaplan-Meier method, and the survival was compared by log-rank test. Cox proportional risk model was used to exclude the potential factors which might influence the survival of patients in the two groups. For subgroups multivariate regression analysis, the adjusted Cox proportional risk model was used to reduce the effects of confounding factors on survival. The adjusted Cox regression analysis considered the age at diagnosis, gender, year of diagnosis, tumor grade, American Joint Committee on Cancer 7th edition (AJCC 7th) T stage, AJCC 7th N stage, tumor size, AFP, chemotherapy, number of tumors, race, marital status, and the type of surgery.

The factors of age at diagnosis, gender, AJCC 7th stage, metastatic organs, race, AFP, fibrosis scores, and chemotherapy were not balanced between the two groups. Thus, propensity score matching (PSM) including all factors analyzed in the study was used to balance the baseline factors. The optimal caliper of the PSM was set as 0.02, and 529 pairs of patients were generated by 1 : 1 ratio matching. After matching, all factors in the two groups were balanced. The statistical analysis was performed using the SPSS 24.0 (IBM, Chicago, IL, USA) and R 3.6.2 software. A *P* value of less than 0.05 was considered statistically significant.

## 3. Results

### 3.1. Characteristics of Patients

A total of 2342 patients were included in the study. Among them, 647 patients received radiotherapy (radiotherapy group), and 1695 did not (nonradiotherapy group). In the radiotherapy group, 467 patients had bone metastases, 63 patients had lung metastases, 8 patients had brain metastases, and 109 patients had multiorgan metastases. In the nonradiotherapy group, 463 patients had bone metastases, 1035 patients had lung metastases, 17 patients had brain metastases, and 180 patients had multiorgan metastases. In the nonradiotherapy group, 746 patients received chemotherapy, 21 patients received ablation (10 patients received radiofrequency ablation and 11 patients received other ablations), and 30 patients received liver resection (Table 1).

### 3.2. Survival Analysis

**Figure 2:**
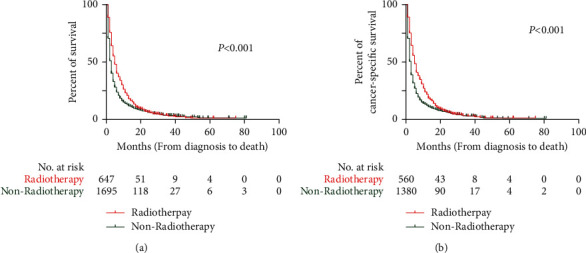
Kaplan-Meier curves of OS and CSS in patients before PSM. (a) Kaplan-Meier curve of OS; (b) Kaplan-Meier curve of CSS.

**Figure 3:**
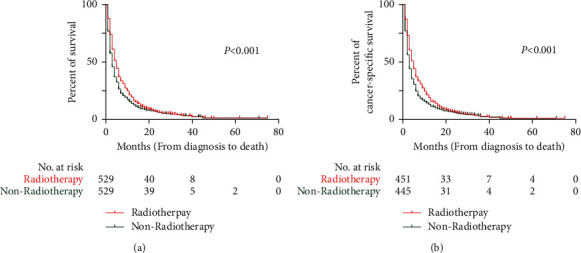
Kaplan-Meier curves of OS and CSS in patients after PSM. (a) Kaplan-Meier curve of OS; (b) Kaplan-Meier curve of CSS.

Before PSM, the median OS (mOS) and median CSS (mCSS) in the radiotherapy group were 5 months (95% CI: 4.5–5.5) and 5 months (95% CI: 4.5–5.5), respectively. These values were longer than those in the nonradiotherapy group (mOS = 3 months, 95% CI: 2.8–3.2; mCSS = 3 months, 95% CI: 2.8–3.2; both *P* < 0.001) (Figure 2). After PSM, the mOS (5 months, 95% CI: 4.5–5.5) and mCSS (5 months, 95% CI: 4.4–5.6) in the radiotherapy group were longer than the mOS (3 months, 95% CI: 2.6–3.4; *P* < 0.001) and mCSS (3 months, 95% CI: 2.6–3.4; *P* < 0.001) in the nonradiotherapy group (Figure 3).

### 3.3. Multivariate Regression Analysis

In the multivariate regression analysis before PSM, female patients, patients with poorly differentiated tumors, patients with the AJCC 7th stage T, and patients with larger tumor size had higher all-cause mortality rate and cancer-specific mortality rate. After excluding potential factors which might influence the survival, patients in the nonradiotherapy group had a higher all-cause mortality rate (HR = 1.277, 95% CI: 1.146–1.424; *P* < 0.001) and cancer-specific mortality rate (HR = 1.315, 95% CI: 1.167–1.481; *P* < 0.001) than patients in the radiotherapy group (Table 2).

### 3.4. Subgroup Analysis

Before PSM, the mOS (6 months, 95% CI: 5.4–6.6) and mCSS (6 months, 95% CI: 5.2–6.8) of patients with bone metastases in the radiotherapy group were longer than the mOS (3 months, 95% CI: 2.5–3.5; *P* < 0.001) and mCSS (3 months, 95% CI: 2.7–3.3; *P* < 0.001) in the nonradiotherapy group. The mOS (5 months, 95% CI: 3.8–6.2) and mCSS (5 months, 95% CI: 3.6–6.4) of patients with lung metastases in the radiotherapy group were longer than the mOS (2 months, 95% CI: 1.8–2.2; *P*=0.011) and mCSS (2 months, 95% CI: 1.8–2.2; *P*=0.043) in the nonradiotherapy group. The mOS (4 months, 95% CI: 3.3–4.7) and mCSS (4 months, 95% CI: 3.3–4.7) of patients with multiorgan metastases in the radiotherapy group were longer than the mOS (2 months, 95% CI: 1.5–2.5; *P*=0.001) and mCSS (2 months, 95% CI: 1.5–2.5; *P* < 0.001) in the nonradiotherapy group. The mOS (5 months, 95% CI: 3.4–6.6) and mCSS (6 months, 95% CI: 3.4–8.6) of patients with fibrosis scores of 0–4 in the radiotherapy group were not statistically significantly longer than the mOS (4 months, 95% CI: 2.6–5.4; *P*=0.868) and mCSS (4 months, 95% CI: 3–5; *P*=0.527) in the nonradiotherapy group. The mOS (6 months, 95% CI: 4.3–7.7) and mCSS (6 months, 95% CI: 4.3–7.7) of patients with fibrosis scores of 5-6 in the radiotherapy group were longer than the mOS (3 months, 95% CI: 2.4–3.6; *P*=0.066) and mCSS (3 months, 95% CI: 2.3–3.7; *P*=0.078) in the nonradiotherapy group (Supplementary Figure 1).

After PSM, the mOS (5 months, 95% CI: 4.4–5.6) and mCSS (6 months, 95% CI: 5.2–6.8) of patients with bone metastases in the radiotherapy group were longer than the mOS (3 months, 95% CI: 2.5–3.5; *P*=0.002) and mCSS (3 months, 95% CI: 2.4–3.6; *P* < 0.001) in the nonradiotherapy group. The mOS (5 months, 95% CI: 3.8–6.2) and mCSS (5 months, 95% CI: 3.6–6.4) of patients with lung metastases in the radiotherapy group were longer than the mOS (3 months, 95% CI: 1.9–4.1; *P*=0.239) and mCSS (3 months, 95% CI: 2.2–3.8; *P*=0.382) of patients in the nonradiotherapy group, but these differences did not reach statistical significance. The mOS (3 months, 95% CI: 2.3–3.7) and mCSS (3 months, 95% CI: 2.3–3.7) of patients with multiorgan metastases in the radiotherapy group were longer than the mOS (2 months, 95% CI: 1.4–2.4; *P*=0.021) and mCSS (3 months, 95% CI: 2.4–3.6; *P*=0.025) in the nonradiotherapy group. The mOS (2 months, 95% CI: 0.5–3.5) and mCSS (8 months, 95% CI: NA) of patients with fibrosis scores of 0–4 in the radiotherapy group were not longer than the mOS (3 months, 95% CI: 1.8–4.2; *P*=0.550) and mCSS (3 months, 95% CI: 1.7–4.3; *P*=0.596) in the nonradiotherapy group. The mOS (6 months, 95% CI: 4.3–7.7) and mCSS (6 months, 95% CI: 4–8) of patients with fibrosis scores of 5-6 in the radiotherapy group were not longer than the mOS (4 months, 95% CI: 2.3–5.7; *P*=0.635) and mCSS (4 months, 95% CI: 2.2–5.8; *P*=0.346) in the nonradiotherapy group, but these differences did not reach statistical significance (Supplementary Figure 2).

Before PSM, the adjusted Cox regression analysis showed that patients with bone metastases in the nonradiotherapy group had a higher all-cause mortality rate (HR = 1.223, 95% CI: 1.062–1.410; *P*=0.005) and cancer-specific mortality rate (HR = 1.326, 95% CI: 1.138–1.545; *P* < 0.001) than patients in the radiotherapy group. Patients with lung metastases in the nonradiotherapy group had a higher all-cause mortality rate (HR = 1.394, 95% CI: 1.053–1.846; *P*=0.02) but not cancer-specific mortality rate (HR = 1.305, 95% CI: 0.961–1.773; *P*=0.088) than patients in the radiotherapy group. Patients with multiorgan metastases in the nonradiotherapy group had higher all-cause mortality rate (HR = 1.387, 95% CI: 1.053–1.827; *P*=0.02) and cancer-specific mortality rate (HR = 1.374, 95% CI: 1.018–1.855; *P*=0.038) than patients in the radiotherapy group. Radiotherapy did not reduce all-cause mortality rate and cancer-specific rate compared to no radiotherapy for patients with fibrosis scores of 0–4 and fibrosis scores of 5-6 (all *P* > 0.05) (Table 3).

After PSM, the adjusted Cox regression analysis showed that patients with bone metastases in the nonradiotherapy group had higher all-cause mortality rate (HR = 1.198, 95% CI: 1.024–1.401; *P*=0.024) and cancer-specific mortality rate (HR = 1.290, 95% CI: 1.089–1.528; *P*=0.003) than patients in the radiotherapy group. Patients with multiorgan metastases in the nonradiotherapy group had higher all-cause mortality rate (HR = 1.438, 95% CI: 1.040–1.989; *P*=0.028) and cancer-specific mortality rate (HR = 1.459, 95% CI: 1.018–2.091; *P*=0.04) than patients in the radiotherapy group. Radiotherapy did not reduce all-cause mortality rate and cancer-specific rate compared to no radiotherapy for patients with fibrosis scores of 0–4 and fibrosis scores of 5-6 (all *P* > 0.05) (Supplementary Table 1).

## 4. Discussion

HCC invading distant organs is considered an advanced stage and predicts poor overall survival [8]. However, only a few investigations have focused on the treatments for patients with advanced HCC. Previous high-quality studies had shown that patients with multiple primary tumor oligometastases had better survival when treated with radiotherapy than when radiotherapy was not used [21, 25, 26]. Moreover, many clinical trials demonstrated that patients with HCC could also receive survival benefits from radiotherapy [22, 27]. However, there was no study focusing on radiotherapy for HCC patients with extrahepatic metastases. Therefore, the present analysis was conducted to compare the survival of HCC patients with extrahepatic metastases who received radiotherapy with that of those that did not receive radiotherapy.

In the current study, HCC patients with extrahepatic metastases who were treated by radiotherapy had longer mOS and mCSS than patients who did not receive radiotherapy; this result was obtained before and after PSM. Previous research documented that the mOS of HCC patients with bone, adrenal gland, or peritoneum metastases who received sorafenib combined with internal radiotherapy was 13.9 months, which was longer than the mOS of 5 months found in the present work [28]. This difference may reflect the use of sorafenib as the first-line treatment of sorafenib, which could prolong the survival time of patients with advanced HCC. Another study on the efficacy of radiotherapy, conducted by Kim and coworkers, included 530 HCC patients with spine, pelvis, rib, or bone metastases. The results demonstrated that the mOS was 5.1 months, which was similar to the mOS found in the current study. In the study of Kim and coworkers, 63% of patients received chemotherapy or sorafenib treatment, a fraction higher than that in the current study (54.5%). However, the patients in Kim et al.'s study did not receive other treatments (ablation or surgery), and, in the current study, 3% of patients were subjected to ablation or surgery, which might explain why mOS values were similar in both studies [29]. In the current study, the mOS and mCSS of patients treated with and without radiotherapy were compared, and the differences in mOS and mCSS between the two groups were similar before PSM and after PSM. This finding implied that patients' death by other reasons did not influence the mOS of all patients.

Univariable regression analysis was not conducted in the current study due to the large sample size of the study; and, in the multivariate regression analysis, after excluding potential confounding factors, the patients who did not receive radiotherapy still had higher all-cause mortality rate and cancer-specific mortality rate, indicating that radiotherapy prolongs the survival of HCC patients with metastases to different extrahepatic organs.

Previous study has documented that patients with metastases to different organs and different liver function status had different survival times [30, 31]. Therefore, subgroup analysis was conducted in the present study to explore whether radiotherapy improved the survival of patients with metastases to different organs and with different fibrosis scores. The Kaplan-Meier analysis showed that the mOS and mCSS of patients with bone metastases and multiorgan metastases were longer in patients treated with radiotherapy than in the nonradiotherapy group. Additionally, the adjusted Cox proportional risk model showed that HCC patients with bone metastases and multiorgan metastases who did not receive radiotherapy had higher all-cause mortality and cancer-specific mortality rates than patients who received radiotherapy. The evaluation of the efficacy of radiotherapy in patients with brain metastases was not conducted here because the number of these patients was small, which might lead to unreliable conclusions. However, in the study, the fibrosis scores of patients did not influence the survival of all patients because the multivariable regression analysis showed that patients with fibrosis scores of 5-6 did not have higher all-cause mortality rate and cancer-specific mortality rate than patients with fibrosis sores of 0–4; and, in the subgroups analysis, radiotherapy did not prolong the survival of patients compared to no radiotherapy, which might show that the liver function of patients might not influence the survival of patients in the current study. However, the fibrosis scores are not a recognized indicator of liver function. Future studies are needed to include Child-Pugh score to confirm the results of the study. The results of subgroup analysis showed that HCC patients with bone metastases and multiorgan metastases could obtain more survival benefits from radiotherapy.

Patients with advanced HCC are recommended to receive atezolizumab plus bevacizumab, sorafenib, and lenvatinib as their first-line treatments and regorafenib, cabozantinib, and ramucirumab as their secondary treatments [8, 32–34]. However, the adverse events of these treatments are high and some parts of patients cannot tolerate it. For these patients, there are no specific treatments recommended. Besides, there are few studies focusing on the systemic therapies on the treatments for HCC patients with extrahepatic metastases. Thus, at present, the results of the study might provide new evidence that HCC patients with extrahepatic metastases could get survival benefits from radiotherapy.

This study has some limitations. First, it was designed as a retrospective study, which might have led to the selection bias. However, selection bias was minimized by conducting PSM. Second, the study did not consider physical condition of patients because the SEER database does not provide this information. Future studies should include these factors to further strengthen the conclusions of the present analysis.

## 5. Conclusion

This study included a large number of HCC patients with extrahepatic metastases, treated or not treated with radiotherapy. The performed analyses documented that radiotherapy-treated HCC patients with bone metastases or multiorgan metastases had longer survival time than patients who were not subjected to radiotherapy. The study provides evidence that can be used clinically to select the best treatment for these patients.

## Figures and Tables

**Table 1:** Baseline characteristics of patients before matching and after matching.

Characteristics	Before matching			After matching		
	Radiotherapy (*N* = 647)	Nonradiotherapy (*N* = 1695)	*P* value	Radiotherapy (*N* = 529)	Nonradiotherapy (*N* = 529)	*P* value
Age at diagnosis			0.009			0.803
30–44	10	54		10	13	
45–59	208	616		178	180	
≥60	429	1025		341	336	
Gender			0.001			0.921
Male	560	1369		449	451	
Female	87	326		80	78	
Years of diagnosis			0.063			0.389
2010–2012	278	801		243	257	
2013–2015	369	894		286	272	
Tumor grade			0.166			0.950
Well differentiated	46	113		39	35	
Moderately differentiated	69	221		60	55	
Poorly differentiated	63	198		52	49	
Undifferentiated	3	16		2	2	
Unknown	466	1147		376	388	
AJCC 7th T stage			0.015			0.799
T0	7	9		6	6	
T1	165	334		115	116	
T2	66	192		59	52	
T3	228	621		189	207	
T4	45	164		36	39	
TX	136	375		124	109	
AJCC 7th N stage			0.195			0.863
N0	434	1075		339	343	
N1	106	327		97	93	
NX	107	293		93	93	
Metastatic organs			<0.001			0.992
Bone	467	463		357	354	
Lung	63	1035		63	65	
Brain	8	17		8	9	
Multiple organs	109	180		101	101	
Tumor size			0.052			0.689
No more than 5 cm	153	325		121	114	
Larger than 5 cm	323	882		258	272	
Unknown	171	488		150	143	
Tumor number			0.186			0.777
1	575	1472		464	467	
≥2	72	223		65	62	
Race			<0.001			0.106
White	472	1074		376	351	
Black	104	317		91	93	
Other/unknown	71	304		62	85	
Marital status			<0.001			0.952
Married	352	767		263	264	
Single	273	825		244	245	
Unknown	22	103		22	20	
AFP			0.002			0.556
Positive	404	1141		338	350	
Negative	75	182		57	60	
Unknown	168	372		134	119	
Fibrosis scores			<0.001			0.447
0–4	34	62		13	19	
5-6	89	242		81	88	
Unknown	524	1391		435	422	
Chemotherapy			<0.001			0.324
Yes	353	746		257	241	
No/unknown	294	949		272	288	
Surgery			0.435			0.881
Ablation	12	21		8	8	
Liver resection	9	30		7	9	
No	626	1644		514	512	

**Table 2:** Multivariable regression analysis for OS and CSS of all patients before PSM.

Characteristics	OS		CSS	
	HR (95% CI)	*P* value	HR (95% CI)	*P* value
Age at diagnosis				
30–44	Reference		Reference	
45–59	1.369 (1.043, 1.797)	0.024	1.244 (0.934, 1.657)	0.136
≥60	1.388 (1.060, 1.818)	0.017	1.290 (0.971, 1.714)	0.079

Gender				
Male	Reference		Reference	
Female	0.870 (0.776, 0.974)	0.016	0.893 (0.788, 1.013)	0.079

Years of diagnosis				
2010–2012	Reference		Reference	
2013–2015	0.985 (0.904, 1.074)	0.734	0.998 (0.908, 1.098)	0.975

Tumor grade				
Well differentiated	Reference		Reference	
Moderately differentiated	1.066 (0.869, 1.306)	0.542	0.989 (0.783, 1.249)	0.928
Poorly differentiated	1.458 (1.185, 1.794)	<0.001	1.390 (1.098, 1.759)	0.006
Undifferentiated	1.231 (0.757, 2.001)	0.401	1.379 (0.799, 2.380)	0.249
Unknown	1.179 (0.992, 1.400)	0.062	1.129 (0.928, 1.374)	0.226

AJCC 7th T stage				
T0	Reference		Reference	
T1	0.801 (0.471, 1.362)	0.141	0.695 (0.364, 1.328)	0.270
T2	1.053 (0.619, 1.792)	0.850	0.918 (0.481, 1.753)	0.796
T3	1.007 (0.590, 1.720)	0.979	0.879 (0.459, 1.682)	0.696
T4	1.020 (0.589, 1.766)	0.973	0.901 (0.465, 1.747)	0.758
TX	0.858 (0.498, 1.477)	0.580	0.732 (0.378, 1.417)	0.355

AJCC 7th N stage				
N0	Reference		Reference	
N1	1.139 (1.017, 1.275)	0.014	1.155 (1.020, 1.307)	0.023
NX	0.911 (0.805, 1.031)	0.141	0.910 (0.793, 1.045)	0.180

Metastatic organs				
Bone	Reference		Reference	
Lung	1.111 (1.000, 1.234)	0.050	1.091 (0.970, 1.226)	0.114
Brain	0.986 (0.660, 1.473)	0.945	0.887 (0.545, 1.442)	0.628
Multiple organs	1.314 (1.145, 1.506)	<0.001	1.301 (1.123, 1.508)	<0.001

Tumor size				
No more than 5 cm	Reference		Reference	
Larger than 5 cm	1.140 (0.988, 1.315)	0.073	1.122 (0.959, 1.312)	0.151
Unknown	1.277 (1.082, 1.506)	0.004	1.258 (1.051, 1.506)	0.012

Tumor number				
1	Reference		Reference	
≥2	0.892 (0.782, 1.017)	0.088	0.591 (0.382, 0.917)	0.019

Race				
White	Reference		Reference	
Black	0.993 (0.887, 1.112)	0.916	0.976 (0.861, 1.106)	0.703
Other/unknown	0.995 (0.882, 1.124)	0.942	1.050 (0.919, 1.199)	0.417

Marital status				
Married	Reference		Reference	
Single	1.035 (0.946, 1.133)	0.453	1.051 (0.952, 1.161)	0.326
Unknown	0.977 (0.807, 1.182)	0.808	0.956 (0.771, 1.185)	0.956

AFP				
Positive	Reference		Reference	
Negative	0.777 (0.673, 0.897)	0.001	0.760 (0.646, 0.894)	0.001
Unknown	0.825 (0.741, 0.918)	<0.001	0.807 (0.716, 0.910)	<0.001

Fibrosis scores				
0–4	Reference		Reference	
5-6	1.002 (0.784, 1.279)	0.990	1.001 (0.762, 1.314)	0.995
Unknown	1.203 (0.964, 1.500)	0.101	1.268 (0.991, 1.623)	0.059

Chemotherapy				
Yes	Reference		Reference	
No/unknown	1.602 (1.466, 1.750)	<0.001	1.617 (1.466, 1.783)	<0.001

Surgery				
Ablation	Reference		Reference	
Liver resection	0.995 (0.589, 1.682)	0.985	1.040 (0.596, 1.817)	0.889
No	2.324 (1.569, 3.442)	<0.001	1.846 (1.218, 2.798)	0.004

Treatment				
Radiotherapy	Reference		Reference	
Nonradiotherapy	1.277 (1.146, 1.424)	<0.001	1.315 (1.167, 1.481)	<0.001

**Table 3:** Adjusted Cox regression analysis for OS and CSS of subgroups. Adjusted for age, gender, race, year of diagnosis, grade, AJCC T stage, AJCC N stage, AFP, chemotherapy, surgery, marriage, tumor size, and tumor number before PSM.

Characteristics	OS		CSS	
	HR (95% CI)	*P* value	HR (95% CI)	*P* value
With bone metastases		0.005		<0.001
Radiotherapy	Reference		Reference	
Nonradiotherapy	1.223 (1.062, 1.410)		1.326 (1.138, 1.545)	
With lung metastases		0.020		0.088
Radiotherapy	Reference		Reference	
Nonradiotherapy	1.394 (1.053, 1.846)		1.305 (0.961, 1.773)	
With multiorgan metastases		0.020		0.038
Radiotherapy	Reference		Reference	
Nonradiotherapy	1.387 (1.053, 1.827)		1.374 (1.018, 1.855)	
Fibrosis scores 0–4		0.630		0.165
Radiotherapy	Reference		Reference	
Nonradiotherapy	1.153 (0.647, 2.053)		2.769 (0.657, 4.797)	
Fibrosis scores 5-6		0.100		0.322
Radiotherapy	Reference		Reference	
Nonradiotherapy	1.255 (0.957, 1.645)		1.328 (0.758, 2.327)	

## Data Availability

The data used in the study are available from SEER database (https://seer.cancer.gov/data/) (accession number: 12577-Nov2019).

## Abbreviations

HCC: Hepatocellular carcinoma
SEER: Surveillance, epidemiology, and end results database
OS: Overall survival
CSS: Cancer-specific survival
PSM: Propensity score matching
AJCC: American joint committee on cancer
EASL: European association for the study of the liver.

## Additional Points

*Guidelines for Methods*.

## Supplementary Materials

Supplementary Materials Table 1: adjusted Cox regression analysis for OS and CSS of subgroups (adjusted for age, gender, race, year of diagnosis, grade, AJCC T stage, AJCC N stage, surgery, marriage, tumor size, and tumor number after PSM). Figure 1: Kaplan-Meier curves of OS and CSS in subgroups before PSM. (A-B) Kaplan-Meier curves of OS and CSS in patients with bone metastases; (C-D) Kaplan-Meier curves of OS and CSS in patients with lung metastases; (E-F) Kaplan-Meier curves of OS and CSS in patients with multiorgan metastases; (G-H) Kaplan-Meier curves of OS and CSS in patients with fibrosis scores of 0–4 and Kaplan-Meier curves of OS and CSS in patients with fibrosis scores of 5-6. Figure 2: Kaplan-Meier curves of OS and CSS in subgroups after PSM. (A-B) Kaplan-Meier curves of OS and CSS in patients with bone metastases; (C-D) Kaplan-Meier curves of OS and CSS in patients with lung metastases; (E-F) Kaplan-Meier curves of OS and CSS in patients with multiorgan metastases; (G-H) Kaplan-Meier curves of OS and CSS in patients with fibrosis scores of 0–4 and Kaplan-Meier curves of OS and CSS in patients with fibrosis scores of 5-6.
