# Supplementary material for: The Efficacy of Radiotherapy in the Treatment of Hepatocellular Carcinoma with Distant Organ Metastasis
Source: J Oncol. 2021 Nov 17;2021:5190611. doi: 10.1155/2021/5190611 (PMC8612773; doi:10.1155/2021/5190611)

**Supplementary materials**

**Table 1** Adjusted Cox regression analysis for OS and CSS of subgroups. Adjusted for age, gender, race, year of diagnosis, grade, AJCC T stage, AJCC N stage, AFP, chemotherapy, surgery, marry, tumor size, and tumor number after PSM

|  | OS |  | CSS |  |
| --- | --- | --- | --- | --- |
| Characteristics | HR (95%CI) | P value | HR (95%CI) | P value |
| **With bone metastases** |  | 0.024 |  | 0.003 |
| Radiotherapy | Reference |  | Reference |  |
| Non-radiotherapy | 1.198 (1.024,1.401) |  | 1.290 (1.089,1.528) |  |
| **With lung metastases** |  | 0.461 |  | 0.645 |
| Radiotherapy | Reference |  | Reference |  |
| Non-radiotherapy | 1.201 (0.738,1.954) |  | 1.142 (0.649,2.010) |  |
| **With multiple organs metastases** |  | 0.028 |  | 0.040 |
| Radiotherapy | Reference |  | Reference |  |
| Non-radiotherapy | 1.438 (1.040,1.989) |  | 1.459 (1.018,2.091) |  |
| **Fibrosis scores 0-4** |  | 0.204 |  | 0.993 |
| Radiotherapy | Reference |  | Reference |  |
| Non-radiotherapy | 3.529 (0.505,24.674) |  | 1.227 (NA) |  |
| **Fibrosis scores 5-6** |  | 0.277 |  | 0.101 |
| Radiotherapy | Reference |  | Reference |  |
| Non-radiotherapy | 1.213 (0.857,1.716) |  | 1.384 (0.939,2.041) |  |

**Figure 1** Kaplan-Meier curves of OS and CSS in subgroups before PSM. (A-B) Kaplan-Meier curves of OS and CSS in patients with bone metastases; (C-D) Kaplan-Meier curves of OS and CSS in patients with lung metastases; (E-F) Kaplan-Meier curves of OS and CSS in patients with multiple organs metastases; (G-H) Kaplan-Meier curves of OS and CSS in patients with chemotherapy; Kaplan-Meier curves of OS and CSS in patients without chemotherapy.


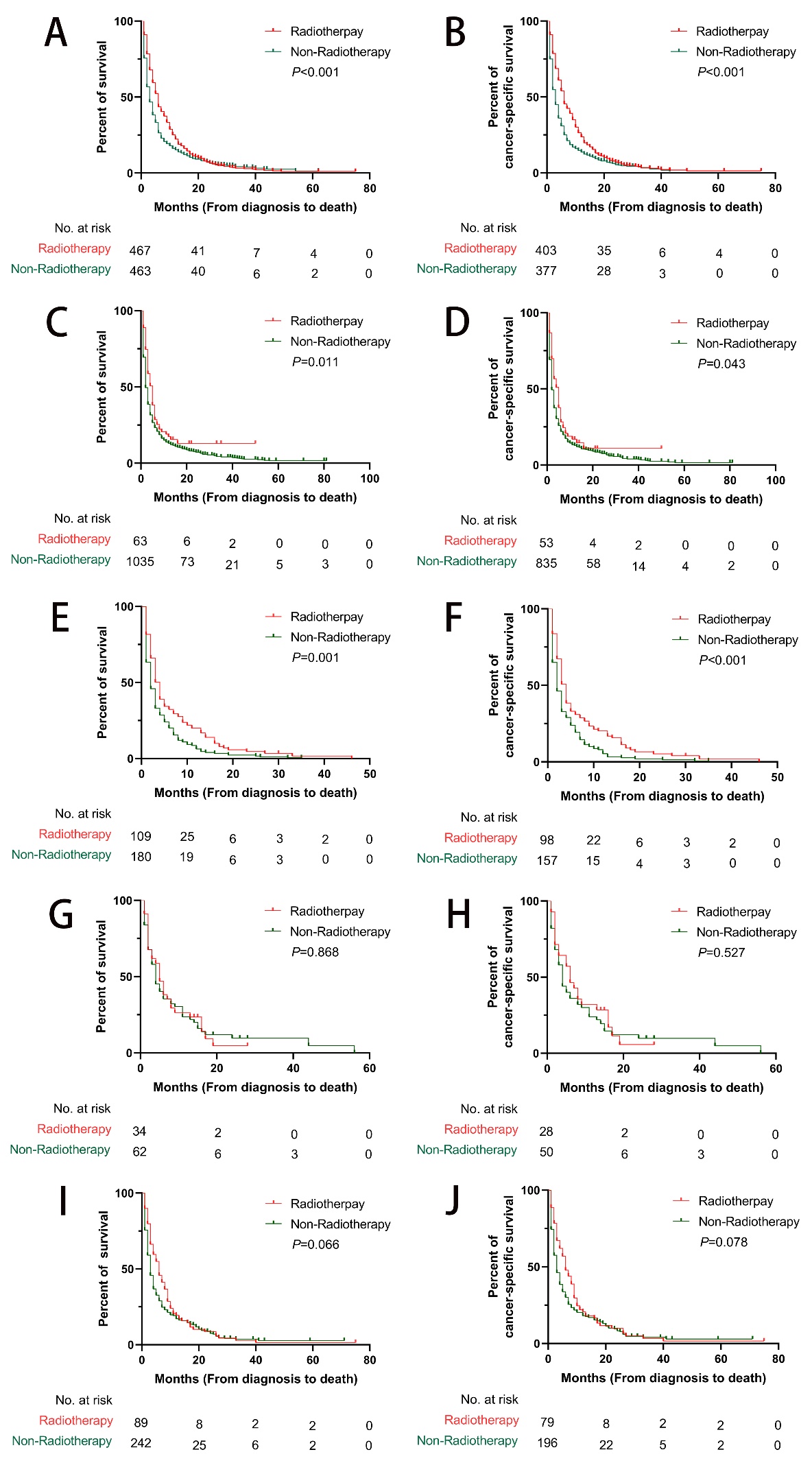


**Figure 2** Kaplan-Meier curves of OS and CSS in subgroups after PSM. (A-B) Kaplan-Meier curves of OS and CSS in patients with bone metastases; (C-D) Kaplan-Meier curves of OS and CSS in patients with lung metastases; (E-F) Kaplan-Meier curves of OS and CSS in patients with multiple organs metastases; (G-H) Kaplan-Meier curves of OS and CSS in patients with chemotherapy; Kaplan-Meier curves of OS and CSS in patients without chemotherapy.


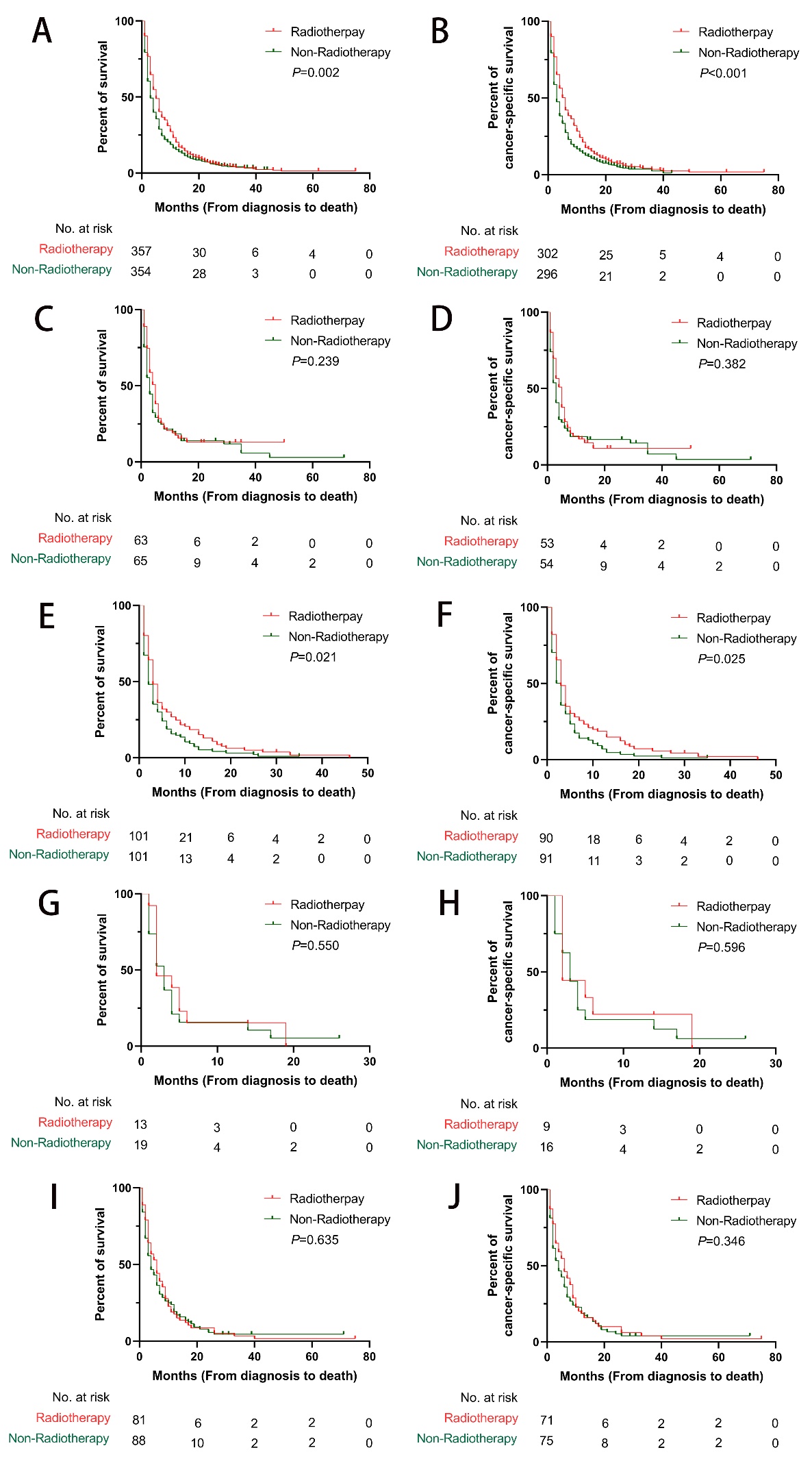

Supplement: Supplementary Materials — Table 1: adjusted Cox regression analysis for OS and CSS of subgroups (adjusted for age, gender, race, year of diagnosis, grade, AJCC T stage, AJCC N stage, surgery, marriage, tumor size, and tumor number after PSM). Figure 1: Kaplan-Meier curves of OS and CSS in subgroups before PSM. (A-B) Kaplan-Meier curves of OS and CSS in patients with bone metastases; (C-D) Kaplan-Meier curves of OS and CSS in patients with lung metastases; (E-F) Kaplan-Meier curves of OS and CSS in patients with multiorgan metastases; (G-H) Kaplan-Meier curves of OS and CSS in patients with fibrosis scores of 0–4 and Kaplan-Meier curves of OS and CSS in patients with fibrosis scores of 5-6. Figure 2: Kaplan-Meier curves of OS and CSS in subgroups after PSM. (A-B) Kaplan-Meier curves of OS and CSS in patients with bone metastases; (C-D) Kaplan-Meier curves of OS and CSS in patients with lung metastases; (E-F) Kaplan-Meier curves of OS and CSS in patients with multiorgan metastases; (G-H) Kaplan-Meier curves of OS and CSS in patients with fibrosis scores of 0–4 and Kaplan-Meier curves of OS and CSS in patients with fibrosis scores of 5-6. [file 5190611.f1.docx]
